# Caloric restriction alleviates aging-related fibrosis of kidney through downregulation of miR-21 in extracellular vesicles

**DOI:** 10.18632/aging.103591

**Published:** 2020-08-27

**Authors:** Jin-rui Liu, Guang-yan Cai, Yi-chun Ning, Jing-chao Wang, Yang Lv, Ya-nan Guo, Bo Fu, Quan Hong, Xue-feng Sun, Xiang-mei Chen

**Affiliations:** 1 Department of Nephrology, Chinese PLA General Hospital, Chinese PLA Institute of Nephrology, State Key Laboratory of Kidney Diseases, National Clinical Research Center of Kidney Diseases, Chinese PLA General Hospital, Beijing 100853, China; 2 Renal Transplant Division, Department of Nephrology, Zhengzhou No. 7 People's Hospital, Zhengzhou 450017, Henan, China

**Keywords:** age, caloric restriction, EMT, extracellular vesicles, miR-21

## Abstract

Glomerulosclerosis and renal interstitial fibrosis occur with the aging kidney. In this study, we examined the expression of miR-21, peroxisome proliferator-activated receptor(PPARα), hypoxia-inducible factor(HIF-1α) in the kidney of 3-month-old rats fed ad libitum (YAL), 24-month-old rats fed ad libitum (OAL) and 24-month-old rats subjected to a 70% calorie-restricted diet for 8 months (OCR). We found long-term caloric restriction (CR) ameliorated aging and aging-related fibrosis. CR ameliorated the increment of miR-21 and HIF-1α, as well as the decrement of PPARα in old ad libitum group. Human proximal tubular cells (HPTCs) presented phenotypes of senescence and epithelial to mesenchymal transition (EMT) under high-glucose conditions, in which senescence occurred earlier than EMT. Senescent cells secreted extracellular vesicles (EVs) which contained miR-21 into the recipient cells. Inhibiting miR-21 of donor cells prevented the occurrence of EMT in recipient cells. In addition, miR-21 induced EMT through targeting PPARα protein and consequently enhancing HIF-1α expression, although other pathways cannot be ruled out. These findings demonstrated that miR-21-containing EVs derived from the senescent cells could facilitate EMT of HPTCs via PPARα-HIF-1α signaling pathway. Long-term caloric restriction and caloric restriction mimetics alleviated aging-related-fibrosis of kidney through downregulation of miR-21.

## INTRODUCTION

Aging in most species associates with impaired adaptive and homeostatic mechanisms. Kidney is a typical target organ of age-associated tissue damage which associates with the high incidences of chronic kidney disease, renal cancer, and renal failure in elderly people [1]. Kidney function decline with aging is characterized with an excessive accumulation and deposition of extracellular matrix (ECM) components which is related to epithelial to mesenchymal transition (EMT) [2]. However, the causal relationship between renal senescence and EMT during kidney aging is not clearly known.

Accumulating evidences showed that senescent cells had adverse effects on the tissue microenvironment. One of the significant changes is senescence-associated secretory phenotype (SASP) that turns senescent fibroblasts into proinflammatory cells that have the ability to promote tumor progression [3]. In fact, SASP might change the composition of extracellular vesicles (EVs). EVs are classified into exosomes, microparticles (MVs), or apoptotic bodies, originating from different subcellular compartments [4]. The size of MVs (100nm ~1000nm in diameter), their lipid composition, irregular shape and density are major characteristics that differentiate them from exosomes(30-100nm). Both exosomes and MVs contain proteins, mRNAs, and micro-RNAs [5]. Zhang et al showed that THP-1-derived MVs could deliver miR-150 into human microvascular endothelial cell line-1 (HMEC-1) cells and enhance exogenous miR-150 expression. This demonstrated cells could secrete miRNAs and deliver them into recipient cells, in which exogenous miRNAs regulate target gene expression and recipient cell function [6].

MicroRNAs serve not only as endogenous regulators of gene expression, but also as potential biomarkers for disorders and secreted factors which mediate cell-cell communication [7]. Accumulating evidences demonstrated miR-21 might be a critical therapeutic target for renal fibrosis [8, 9]. However, the mechanisms are not clearly known. Chau BN et al showed that miR-21 contributed to fibrogenesis and epithelial cells injury in the kidney of unilateral ureteral obstruction (UUO) and unilateral ischemia reperfusion injury (IRI) models, in which peroxisome proliferator activated receptor-α (PPARα), a direct target of miR-21, was involved [10]. PPARα expression was uniformly localized in cytoplasm of cortical tubular epithelial cells in normal kidneys. In obstructed kidneys, PPARα expression was significantly reduced [11]. But its role in kidney fibrosis was unknown. In addition, Ichihara S et al showed that fenofibrate, an activator of PPARα reduced cardiac fibrosis through modulation of c-Jun/HIF-1α signaling [12]. In this study, we want to investigate whether miR-21 secreted from senescent cells induces EMT by inhibiting its target genes PPARα and activating HIF-1α, as well as the protective effects of long-term caloric restriction and caloric restriction mimetics on aging-related-fibrosis of kidney.

## RESULTS

### Long-term CR reduced senescent changes and fibrosis in aged rat kidney

**Figure 1:**
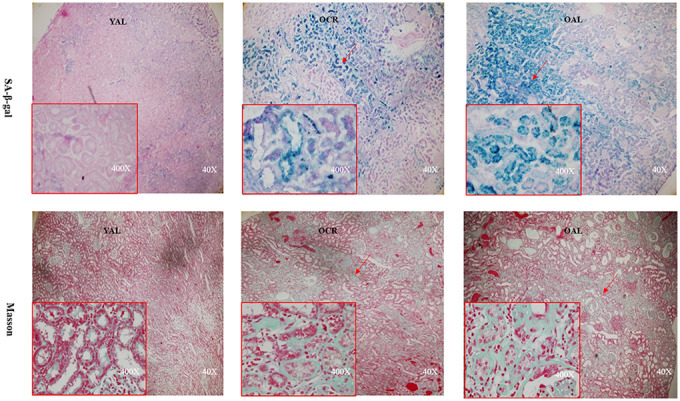
**Renal cortex from three groups.** The sections were stained with SA-β-gal and Masson’s trichrome staining for the assessment of kidney morphology changes (× 40/ 400 magnification).

**Figure 2:**
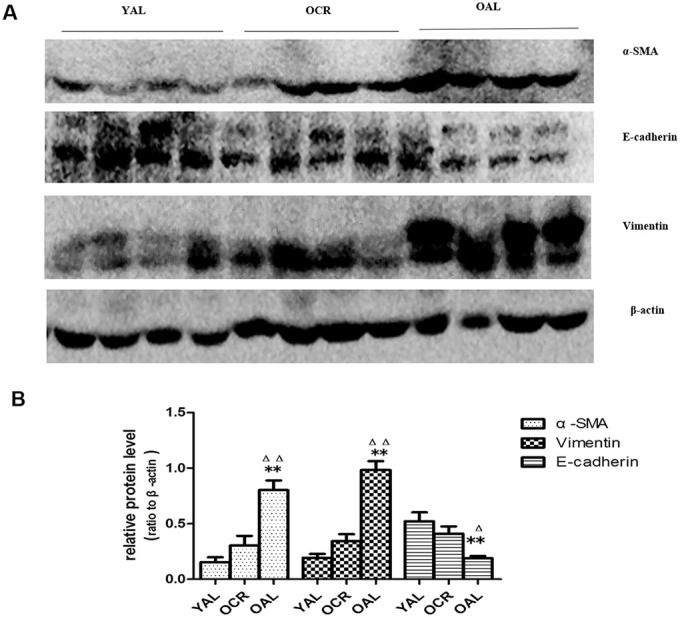
**Expression of EMT biomarker in the kidneys of three groups.** (**A**) Western blot results for α -SMA, E-cadherin, vimentin protein. (**B**) Quantitative analysis of band density for α-SMA,E-cadherin, vimentin protein. Data were presented as means ± SD (n=6). * p < 0.05, ** p < 0.01 (OAL vs YAL); ^Δ^ p< 0.05, ^ΔΔ^ p < 0.01 (OAL vs OCR).

SA-β-gal staining is a robust biomarker of mammalian aging which can be detected in senescent cells and tissues [13]. Kidney aging is often accompanied by renal fibrosis which is associated with myofibroblasts caused by epithelial-to-mesenchymal transition (EMT) [14]. EMT is characterized as an increased expression of α-smooth muscle actin(α-SMA) and vimentin and a reduced expression of E-cadherin in epithelial cells. The expression of SA-β-gal was detected in the kidney of 3-month-old rats fed ad libitum (YAL), 24-month-old rats fed ad libitum (OAL) and 24-month-old rats subjected to a 70% calorie-restricted diet for 8 months (OCR). The positive SA-β-gal staining of kidney was markedly higher in OAL group than that in YAL group. Long-term CR decreased SA-β-gal staining in OCR group (Figure 1). Masson’s trichrome staining was used for collagen fibers detection. Positive areas of collagen fibers were higher in OAL group than that in OCR and in YAL group (Figure 1). The expressions of α-SMA and vimentin were elevated and E-cadherin was declined in kidneys of OAL compared to those in YAL and OCR by immunohistochemistry staining and western blot analysis, respectively (Supplementary Figure 1 and Figure 2). These findings indicated that the degree of fibrosis was correlated with kidney aging. Long-term CR ameliorated renal senescence and renal fibrosis. In OCR group, body weight and kidney weight of the rats were lower than those in OAL group (322.3±12.8g vs 499.5±24.2g and 2.22±0.11g vs 2.86±0.05g, respectively).

### HPTCs senescence and EMT occurred in response to high glucose treatment

To explore the relationships between renal cell senescence and EMT, HPTCs were cultured under high-glucose conditions in vitro. High glucose (33mM) was used to stimulate HPTCs at different times (0h, 6h, 12h, 24h, 48h, 72h), the sequential occurrence of senescence and EMT was detected according to each specific marker. The SA-β-gal staining and senescence marker P53 and P21 were significantly increased at 24h in the high-glucose group. Immunofluorescence staining and western blot analysis showed EMT markers were significantly changed at 72h (Supplementary Figure 2A–2H). These results indicated that high glucose induced HPTCs senescence and EMT. The senescent phenotype change happened earlier than that of EMT.

**Figure 3:**
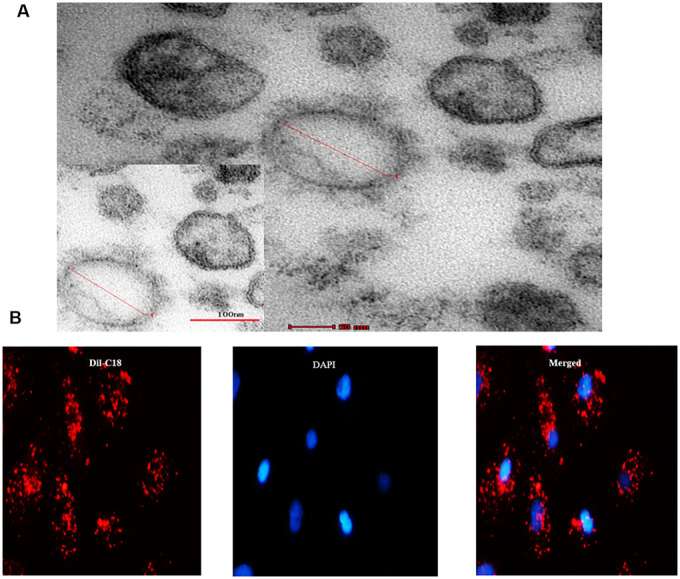
**MVs derived from cell culture media.** (**A**) Transmission electron microscopy (TEM) micrograph of EVs isolated from cell culture media. (**B**) Microscopy image showed the internalization of fluorescently labeled EVs into recipient HPTCs with Dil-C18 (red).

Consequently, we investigated whether the senescent cells secrete SASP factors into neighboring recipient HPTCs to promote EMT. EVs were obtained from cell culture medium by differential centrifugation technique. Under transmission electron microscope, EVs appeared as clusters of vesicles (approximately 50 to 1000 nm in diameter), in which SMVs were surrounded by a double-layer membrane (100 to 1000nm) and exsomes were surrounded by a single-layer membrane (30 to 100nm) (Figure 3A). Next we investigated whether EVs could enter into the recipient cell. EVs were labeled with Dil-C_18_ and then applied to treat recipient HPTCs (Figure 3B). Dil-C_18_ labeled EVs rapidly went into the recipient HPTCs at 37°C after 6 hours. These results suggested that recipient HPTCs resulted from Dil-C18 internalization into targeted cells. EVs were probably the major carriers for cell-cell communication between tubular epithelial cells.

### Long-term caloric restriction down-regulated miR-21 excretion in renal tissues and urines

**Figure 4:**
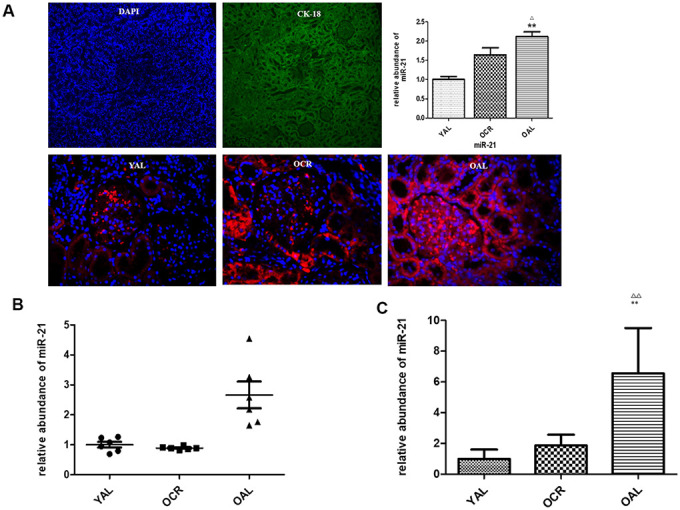
**miR-21 expression in rat kidney tissue detected by FISH and qPCR.** (**A**) The tubular epithelial cells were marked by DAPI and Cytokeratin 18 (CK-18);In situ hybridization of miR-21 in renal biopsies of rats in YAL and OAL; Quantification of in situ hybridization results. **P<0.01. (**B**) Comparison of miR-21 level in tissues. Black triangles indicated the irrelative miR-21 abundance. The black lines represented the means of miR-21 abundance. Statistical significance was present between OAL, YAL and OCR groups. (**C**) Comparison of miR-21 level in urine EVs. qRT-PCR analyses of miR-21 by relative quantification respectively. ** p < 0.01 (YAL vs OAL); ^ΔΔ^p < 0.01 (OCR vs OAL).

MiR-21 is considered as a key mediator in kidney fibrosis. It also served as a potential biomarker of kidney fibrosis [15]. The following experiment is to determine whether miR-21 in extracellular vesicles were secreted by tubular epithelial cells and whether long-term caloric restriction could reduce its expression. Firstly, FISH was applied to localize miR-21 in kidney tissues of YAL and OAL. The results showed miR-21was hardly detectable in the kidney of YAL/OCR, the expression of miR-21 was markedly increased in the kidney of OAL (Figure 4A), There are significant differences between the two groups (Figure 4A).

Secondly, qPCR analysis confirmed the expression of miR-21 was decreased in kidney tissues of YAL and OCR groups comparing to OAL group (Figure 4B). To further verify whether the miR-21 in tubular cells could be secreted in EVs, the miR-21 levels of EVs in urines of the three groups were examined. EVs were obtained from a 2 mL volume of urine according to the previous method. Compared with OAL group, miR-21 expression of urine EVs was down-regulated in YAL and OCR groups (Figure 4C), which suggested caloric restriction reduced the expression of miR-21 in EVs.

Over-expression of miR-21 in HPTCs and EVs from conditioned medium induced by high glucose were ameliorated by resveratrol

Resveratrol shares many similar biological effects induced by caloric restriction. To explore whether miR-21 over-expression in high glucose was ameliorated by resveratrol, HPTCs were cultured under high glucose (HG) and HG combined resveratrol (HG+RSV) conditions in vitro. Cell senescence in HG group was improved in HG+RSV group confirmed by western blot and SA-β-gal staining analysis (Supplementary Figure 3A–3D). To establish the role of miR-21 in senescence of HPTCs, miR-21 levels in normal culture condition (CON), HG and HG+RSV groups were compared by qRT-PCR. Through detecting its expression in HPTCs and microvesicles from culture medium, the level of miR-21 in HG group were substantially higher than that in CON groups, while HG+RSV group had lower level compared with HG (Supplementary Figure 3E, 3F). These results suggested miR-21 participated in aging-related kidney fibrosis.

### miR-21 containing EVs from senescent cells promoted HPTCs phenotype transition

**Figure 5:**
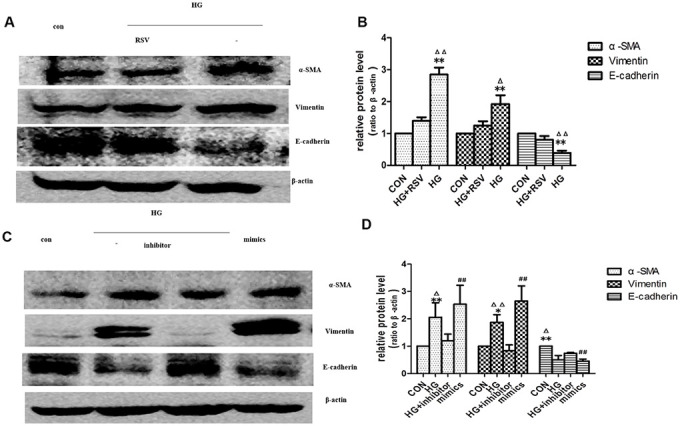
**Exogenous miR-21 was delivered by EVs into recipient tubular cells and promoted EMT.** (**A**) Western blot analysis showed that resveratrol inhibited senescence of recipient cells, blocking EVs from high-glucose conditioned medium induced decline of E-cadherin and increase of α-SMA and vimentin. (**C**) Western blot analysis showed that inhibition of miR-21 by transfection of miR-21 inhibitor in recipient cells blocked EVs from high-glucose conditioned medium induced decline of E-cadherin and increase of α-SMA and vimentin. Upregulation of miR-21 by miR-21 mimics transfection in recipient cells promoted EVs-induced inhibition of E-cadherin and inductions of α-SMA and vimentin expression. (**B**, **D**) Quantitative analysis of band density for the proteins. The protein expression data are presented as the mean ± SD. * p < 0.05 (HG vs CON); ^Δ^p < 0.05 (HG vs HG+inhibitor). ^#^ p < 0.05 (mimics vs CON); **/^ΔΔ^/^##^ p < 0.01.

EV-mediated delivery of small molecules was involved in cell-cell communication [16]. miRNAs played an important role in many biological processes. Studies showed that endogenous plasma miRNAs existed in a form that was resistant to plasma RNase activity [17]. So endogenous plasma miR-21 was considered to have stability for packaging by EVs. To determine whether exogenous miR-21 secreted by senescent cells was delivered by EVs and promoted tubular phenotype transition, EVs obtained from different conditioned media by sequential centrifugation were used to treat recipient cells. miR-21 containing EVs secreted by high-glucose induced premature senescent cells induced HPTCs phenotype transition, which was partially reversed by resveratrol (Figure 5A, 5B). After transfection of miR-21 mimic or inhibitor, the EVs were then collected to treat recipient cells (Figure 5C, 5D), in which HPTCs phenotype transition was induced or inhibited, respectively. These data confirmed the effect of miR-21 containing EVs on the promotion of HPTCs phenotype transition.

### miR-21 induced EMT via targeting PPARα protein

**Figure 6:**
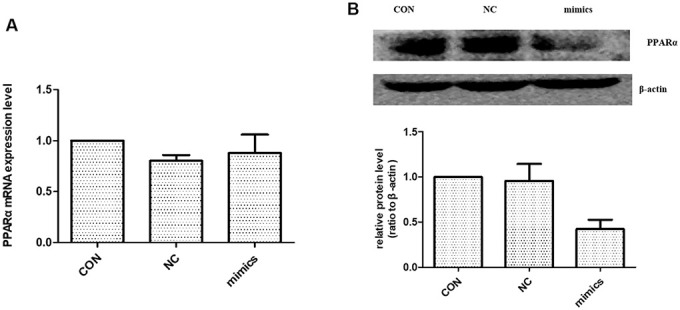
**The effect of miR-21 on PPARα expression.** (**A**) PPARα mRNA expression level were detected with Real-time PCR. (**B**) PPARα protein expression level was detected with Western blot. CON: normal culture condition; NC: miR negative control; Mimics: miR-21 mimics. * p<0.01(mimic group vs CON group), n=3.

Previous studies identified a high-level expression of PPARα in kidney. PPARα is mainly localized in proximal tubules, which emphasizes the importance of fatty acid metabolism in renal physiology [18]. According to the literature, miR-21 can regulate PPARα level by targeting its 3′-UTRs [10, 19]. To test whether miR-21 directly regulates PPARα in HPTCs, HPTCs were transfected with miR-21 mimics. Western blot showed the expression of PPARα protein was increased (Figure 6B). However, there was no detectable alteration in PPARα mRNA levels after miR-21mimics transfection (Figure 6A). These results confirmed that miR-21 regulates PPARα expression by inhibiting the translation, but not destabilization of its mRNA.

### miR-21 amplified HPTCs phenotype transition through enhancing HIF-1α signaling

**Figure 7:**
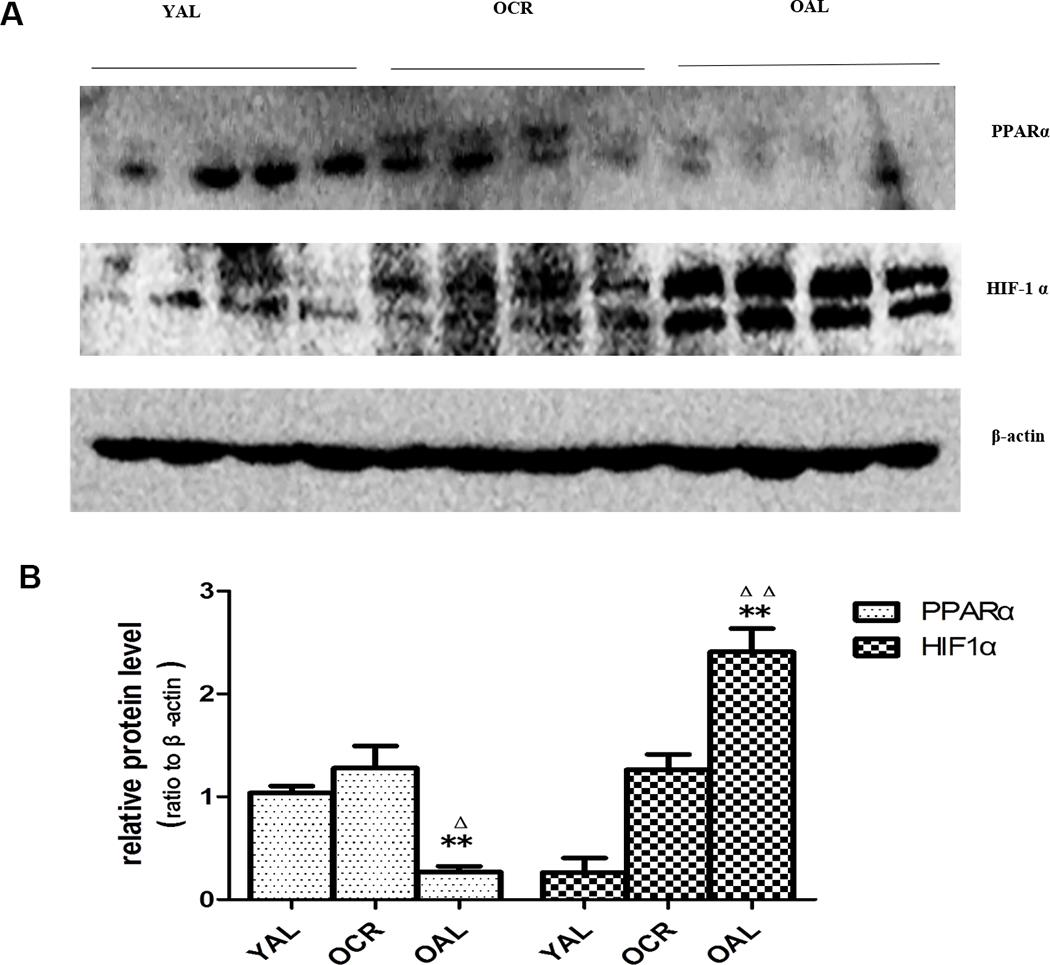
**Expression of PPARα and HIF-1α in the kidneys of three groups.** (**A**) Western blot results for PPARα and HIF-1α protein. (**B**) Quantitative analysis of band density for PPARα and HIF-1α. Data are presented as mean ± SD (n=4). * p < 0.05 (OAL vs YAL); ^Δ^p < 0.05 (OCR vs OAL). **/^ΔΔ^p < 0.01.

As previously mentioned, the expression of miR-21 was increased significantly in kidney tissue of OAL compared to YAL and decreased significantly in OCR compared to OAL. To explore the relationships among miR-21, PPARα and HIF-1α, the expressions of PPARα and HIF-1α in kidney tissues of the three groups were detected by western blot. The total PPARα protein expression was markedly lower and the total HIF-1α protein expression was significantly higher in OAL group than in the YAL and OCR groups (Figure 7), which demonstrated that PPARα was suppressed, but HIF-1α was increased in aging kidney.

**Figure 8:**
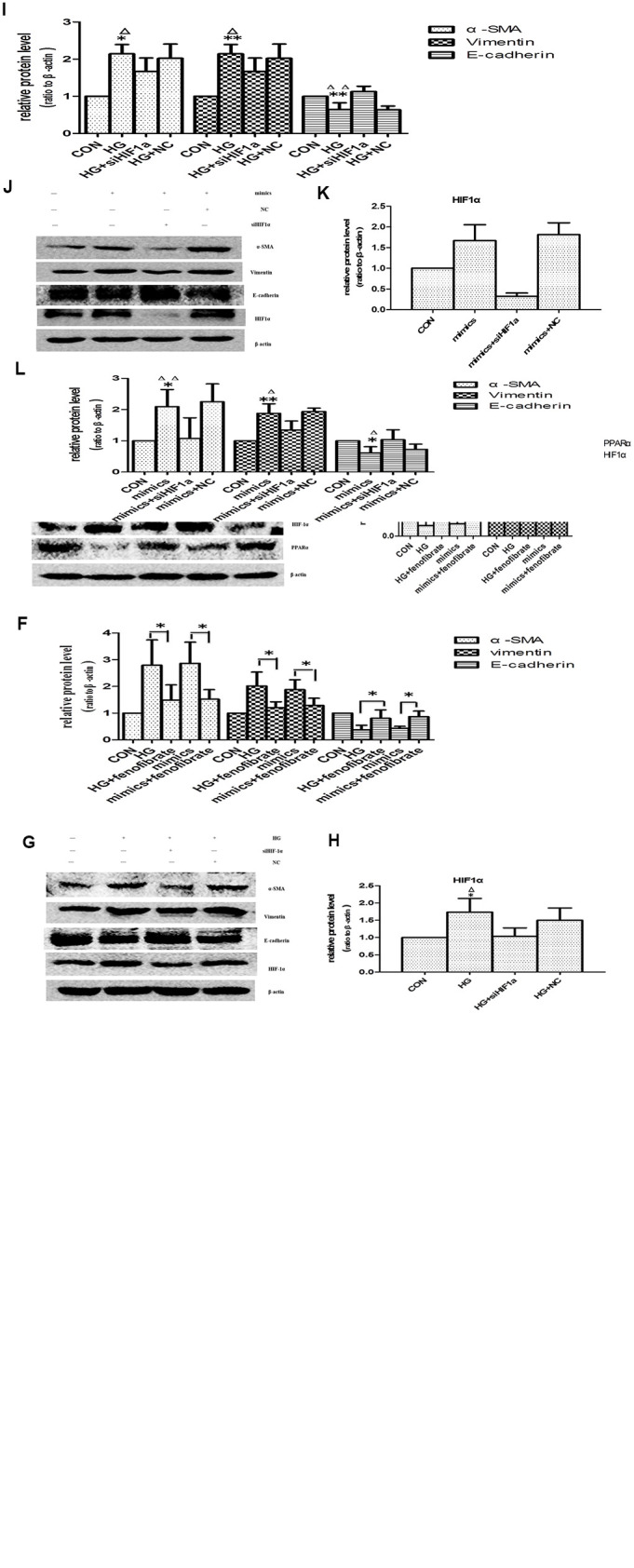
**Effects of miR-21 on PPARα/HIF-1α signaling.** (**A**) Cells incubated under normal glucose (CON), high glucose (HG), miR-21 mimics, HG+miR-21 inhibitor, miR negative control (NC), and HG+NC for 48h were harvested for Western blot analysis of EMT marker and HIF-1α signaling. (**B**, **C**) Quantitative analysis of band density for E-cadherin, α-SMA, vimentin and HIF-1α. Data are presented as mean ± SD. * p < 0.05 (HG vs CON); ^Δ^p < 0.05 (HG vs HG + inhibitor); ^#^ p < 0.05 (mimics vs CON); **/^ΔΔ^/^##^ p<0.01. (**D**) Cells incubated under CON, HG, miR-21mimics, HG+fenofibrate, miR-21mimics+fenofibrate for 48h were harvested for Western blot analysis of EMT marker and PPARα/HIF-1α signaling. (**E**, **F**) Quantitative analysis of band density for E-cadherin, α-SMA, vimentin, PPARα and HIF-1α. Data are presented as mean ± SD. * p < 0.05 (HG vs CON); ^Δ^p < 0.05 (HG vs HG+ fenofibrate); ^#^ p < 0.05 (mimics vs CON); ^$^ p < 0.05 (mimics vs mimics+ fenofibrate). **/^ΔΔ^/^##^/^$$^ p<0.01. (**G**) HPTCs treated with CON, HG, HG+ siHIF-1α, and HG+ si-con (NC) for 48 h were harvested for Western blot analysis of EMT marker and HIF-1α signaling. (**H**, **I**) Quantitative analysis of band density for E-cadherin, α-SMA, vimentin and HIF-1α. Data are presented as mean ±SD. *p < 0.05 (HG vs CON); ^Δ^p < 0.05 (HG vs HG+ siHIF-1α). **/^ΔΔ^p<0.01. (**J**) HPTCs treated with CON, miR-21mimics, miR-21mimics+siHIF-1α, and miR-21mimics+si-con (NC) for 48 h were harvested for Western blot analysis of EMT marker and HIF-1α signaling. (**K**, **L**) Quantitative analysis of band density for E-cadherin, α-SMA, vimentin and HIF-1α. Data are presented as mean ± SD. * p < 0.05 (mimics vs CON); ^Δ^p < 0.05 (mimics vs mimics+siHIF-1α). **/^ΔΔ^p<0.01.

The change pattern of miR-21 was consistent with HIF-1α, so we performed next experiment to focus on whether miR-21 induced EMT through enhancing HIF-1α expression. The effects of miR-21 on EMT were determined by transient transfection of miR-21 mimic and miR-21 inhibitor. After transfection of miR-21 mimic, HPTCs displayed loss of E-cadherin, induction of α-SMA, vimentin and HIF-1α, which were similar to the findings induced by high-glucose treatment (Figure 8A–8C). The over-expressions of α-SMA, vimentin and HIF-1α, and the decline of E-cadherin by high-glucose were diminished by miR-21 inhibitor transfection. These data confirmed miR-21 enhanced HPTCs phenotype transition and HIF-1α expression.

PPARα regulates glucose homoeostasis, lipid metabolism, and interacts negatively with nuclear factors (AP1 and NF-κB), which lead to inhibition of some pro-inflammatory genes [10]. In addition, Chau BN et al. shown that miR-21 promoted kidney fibrosis by silencing PPARα [10]. However, the effects of PPARα on aging-related-fibrosis of kidney and HPTCs phenotype transition have not been fully determined. Fenofibrate is a kind of specific peroxisome proliferator-activated receptor alpha (PPARα) agonists [20]. Here fenofibrate was used to increase PPARα expression. Activation of PPARα suppressed HIF-1α expression and prevented EMT induced by high-glucose or miR-21(Figure 8D–8F). Therefore, PPARα plays an important role in aging-related kidney fibrosis.

The expression of HIF-1α was increased in aged kidney or high-glucose induced premature cell senescence. To explore whether HIF-1α mediates HPTCs phenotype transition induced by miR-21, siRNA-HIF-1α was successfully designed. HPTCs were transfected with either siRNA-HIF-1α or irrelevant siRNA-con. After 48h, HIF-1α expression of HPTCs was detected by western blot analysis. HIF-1α-transfected HPTCs resulted in a dramatic decreased expression of HIF-1α protein, while non-transfected control and siRNA-con did not (Figure 8G, 8H, 8J, 8K). We further examined how the EMT was regulated by HIF-1α inhibition. After treatment with HG or miR-21 mimics, HPTCs transfected with siRNA-HIF-1α showed significant up-regulation of E-cadherin and down-regulation of α-SMA and vimentin compared with non-transfected control cells (Figure 8G, 8I, 8J, 8L).

These results mentioned above suggested that miR-21 promoted EMT specifically dependent on upregulation of HIF-1α signaling pathway, which attributed to the target suppression of PPARα protein by miR-21.

## DISCUSSION

Aging was a systematic process that affects all organs. Kidney showed structural and functional changes with aging. The pathological characteristics of aged kidneys consisted of glomerular sclerosis and tubulointerstitial fibrosis [21]. Tubulointerstitial fibrosis was considered as a central event in the progression of ESRD. Therefore, slowing or reversing aging-related renal fibrosis may be a therapeutic strategy for protection of kidney function.

Calorie restriction had been shown to increase lifespans of many species, including yeast, fish and mammals. In this study, we focused on the protective effect of long-term (8-month) CR on kidney of aged rats. Our results showed that long-term CR markedly attenuated renal aging and associated renal fibrosis. It is unclear whether there was a cause-and-effect relation between kidney aging and kidney fibrosis. Therefore, we investigated the relationship in senescent HPTCs induced by high glucose. Classically, cellular senescence, including human cells, involves the p53/p21 and p16^INK4a^/pRb pathways. p53 and pRb were the main transcriptional regulators, while p21 was a downstream effector of p53 and p16^INK4a^ was a positive upstream regulator of pRb [22]. By western blot analysis, we confirmed that HPTCs senescence developed at 24h. Later, HPTCs epithelial–mesenchymal transition appeared at 48h. These data indicated that the senescence phenotype of HPTCs occurred earlier than EMT. Previous studies demonstrated epithelial cell cycle arrest in G2/M mediated kidney fibrosis [23], while cellular senescence was determined by G0/G1 cell cycle arrest [24]. As senescence and EMT could not occur simultaneously within the same cell, we hypothesized that senescent cells may promote the initiation and development of EMT in neighbor cells.

It has been demonstrated that senescent cells expressed a senescence-associated secretory phenotype (SASP), which has powerful paracrine activities including EMT stimulation owing to some proteins or other biological factors [24]. microRNAs provided critical functions in a variety of biological process, for example, cellular proliferation, apoptosis, insulin secretion and so on. microRNAs could be used as biomarkers of many diseases [25]. microRNAs in EVs can avoid being digested by RNA enzymes in microenvironment. EVs could be used as messengers for cell-cell communication. We confirmed this through transmission electron microscopy and Dil-C18. As previous studies suggested that upregulation of miR-21 could promote renal fibrosis [26], we speculated that miR-21 might play an important role in aging related kidney fibrosis. We found an increased level of miR-21 in OAL and long-term CR decreased them. To further test whether long-term CR could affect miR-21 expression, EVs of urine supernatant in the three groups were extracted and compared, which was consistent with the findings on kidney tissues. Then, Sirt1 agonists resveratrol was used to mimic CR in vitro, miR-21 was significantly higher in HG group than in the control group. HG+RSV groups decreased miR-21 levels in cells or EVs. At present, more attentions have been paid on the function and mechanism of miRNA in EVs. Zhang G et al. had shown that miR-21 derived from tumor cells was packaged into EVs, and then directly delivered to endothelial cells. miR-21 activated JAK-STAT pathway to promote endothelial cell migration and angiogenesis [27]. In this study, EVs obtained from different conditioned media of donor cells were transferred into recipient cells. miR-21 was activated or reduced through transfected with miR-21 mimics or inhibitor. EMT was induced significantly in HG and miR-21 mimics groups, comparing with Con, HG+RSV or HG + inhibitor groups. These results indicated that miR-21 in EVs from senescent cells might play a significant role in promoting EMT of the neighbor cells. Targeting miR-21 might be beneficial for improving aging-related renal fibrosis. As downregulation of miR-21 expression prevented cell proliferation [26, 28], it is necessary to investigate the mechanism of miR-21 in renal fibrosis.

PPARα, a member of the steroid hormone family of intracellular receptors that regulates a number of lipid oxidation, glycose metabolism, inflammation and metabolism pathways [10]. As the PPAR*α*-targeting microRNA, miR-21 efficiently decreases PPAR*α* expression when ectopically expressed in renal epithelial cells [29]. But the functions of PPARα have been controversial. On one hand, overexpression of PPARα leads to irreversible damage of variable organs. For example, activation of PPAR-α in reperfusion of ischemic myocardia cells leaded to elevated fatty acid oxidation, decreased glucose utilization and cardiac dysfunction [30, 31]. On the other hand, PPARα, as the major protein of lipid metabolism, had protective effects in prevention of nephritic fibrosis. BAY PP1, a novel PPAR-α agonist, significantly reduced tubulointerstitial fibrosis, TGF-β expression, and proliferation of interstitial fibroblasts in unilateral ureteral obstruction and 5/6 nephrectomy models [11]. PPARα had shown the protection in ameliorating cardiac fibrosis by inhibiting TGF-β signaling [32]. We confirmed that the expression of PPARα was suppressed, and HIF-1α were induced in aged kidney. Furthermore, fenofibrate, as a specific agonist of PPARα, could alleviated the occurrence of EMT induced by high glucose or miR-21 through suppressing HIF-1α expression. Actually, studies have confirmed that activation of PPARα suppressed HIF-1α signaling in human breast (MCF-7) and ovarian (A2780) cancer cells under hypoxia [33]. Although HIF-1α was found to be involved in fibrosis, the relationship among miR-21, PPARα and HIF-1α in aging-related renal fibrosis has not been reported. By transfecting with siRNA-HIF-1α, miR-21mimics, miR-21inhibitor, and using PPAR-α agonist, the results indicated that miR-21 mediated SASP-related epithelial to mesenchymal transition via activation of HIF-1α by targeting PPARα.

**Figure 9:**
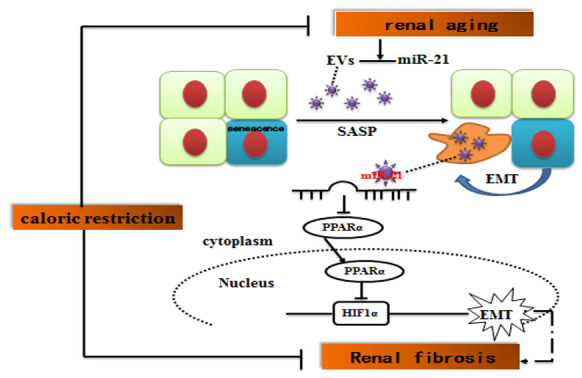
**Suggested molecular mechanisms underlying caloric restriction-mediated the prevention of aging-related renal fibrosis.** The overexpression of miR-21 in EVs could result in downregulation of PPARα, and then promoted activation of HIF-1α inducing tubular phenotype transition in aged kidney. Caloric restriction may prevent the above-mentioned process.

In summary, our study demonstrated that miR-21-containing EVs derived from the senescent cells could facilitate EMT of HPTCs. miR-21 induced EMT through targeting PPARα protein and consequently enhancing HIF-1α expression, although other pathways cannot be ruled out. Long-term caloric restriction and caloric restriction mimetics alleviated kidney aging and aging-related-fibrosis through downregulation of miR-21 (Figure 9). These results are helpful to understand how SASPs induce epithelial to mesenchymal transition due to EVs, and to explore CR as a potential nutritional intervention to delay renal senescence and age-related renal fibrosis.

## MATERIALS AND METHODS

Fetal bovine serum (FBS) were supplied by Invitrogen (Grand Island, NY); F12 and Dulbecco’s modified Eagle’s medium (DMEM) were supplied by GIBCO (Gaithersburg, MD); Penicillin, streptomycin, dimethyl sulfoxide (DMSO) and TRIzol reagent were bought from Invitrogen (Carlsbad, CA); Leupeptin, aprotinin, RIPA, phenylmethylsulfonylfluoride were obtained from Sigma (St Louis, MO); CASEIN was bought from Vector (Burlingame, CA); Anti-E-cadherin, anti-vimentin, anti-α-SMA were obtained from Abcam (Cambridge, MA); Anti-HIF-1α, anti-PPARα, anti-P53, anti-P21 were from Proteintech (Chicago, CA).

### Human PTCs isolation and incubation

Isolation and incubation of primary human PTCs were performed as previously described [34]. Briefly, HPTCs were isolated from kidney of patients who suffered from nephrectomy and cultured in DMEM/F12 (DMEM:F12=1:1) containing 10% FBS, EGF 10ng/mL, transferrin 5 μg/mL, insulin 5 μg/mL, 100 μg/mL penicillin and 100 μg/mL, streptomycin at 37°C. The third passages were used in this study. In order to explore the effect of high glucose on HPTCs, serum-deprived cells were separated into normal glucose control [cultured in 5.5 mM D-glucose (NG)], high-glucose group [cultured in 33 mM D-glucose (HG)]. Incubations were performed in a humidified 37°C, 5% CO_2_ incubator (Sanyo Corporation).

### Animals and calorie restriction

Fisher 344 rats (3 month, n=6; 16month, n=12) were purchased from the Vital River Laboratory Animal Technology, Co, Ltd (Beijing, China) and were used at the Chinese PLA General Hospital. All experiments involving animals were kept under a specific pathogen-free condition: 22±1°C, 40% humidity, 12:12h light/dark cycle, one male per cage, and free access to water. Young rats (YAL) were fed on standard laboratory food, containing 23.0% crude protein, 10.0% water, 5.0% crude fat, 51.0% crude carbohydrate, 7.0% crude ash, 4.0% crude fiber. After 16 months of age, the 12 Fisher 344 rats were divided into 2 groups: OAL and OCR (n=6 per group). During the following 8 months, rats of the OCR group were fed with food corresponding to 70% of the amount of food consumed by the OAL group. Food consumption was measured every 2 weeks, and the results were used to calculate the daily food intake. CR diets were enriched in vitamins, minerals, salts and so on which the rats of caloric restriction were not nutrient-deficient or salt-deficient compared to the control animals. Finally, the 3-month-old (YAL) rats and the 24-month-old (OAL, OCR) rats were sacrificed. Rats were anesthetized via intraperitoneal injection of sodium pentobarbital (40mg/kg). Before sacrificed, rats were housed in individual metabolic cages to collect urine for 24h. The kidney tissues of rats were removed and perfused with phosphate-buffered saline (PBS; pH 7.4) to remove remaining blood. A portion was immersed into OCT compound (Tissue-Tek; Sakura Finetek, Torrance, CA, USA) for immuno-histochemistry staining. The remaining tissue was immediately frozen in liquid nitrogen and stored at 80°C for Western blot analysis.

### Immunohistochemistry

The kidneys were fixed in 10% formaldehyde overnight at 4°C and processed for paraffin-embedding following standard procedures. Sections were prepared at 2 mm thicknesses. For immunohistochemical analysis, some tissue sections were subjected to antigen retrieval by microwaving or autoclaving for 11 min in 10 mM sodium citrate buffer [pH 6.0]. Endogenous peroxidase activity was blocked by incubation with 3% hydrogen peroxide for 15 min. After PBS washing, sections were incubated with 1.5% normal goat serum for 30 min, followed by incubation with mouse monoclonal anti-E-cadherin (1:50; Cambridge, MA) and rabbit polyclonal anti-α-SMA (1:100, Cambridge, MA) overnight at 4°C. After three washes with PBS, the samples were incubated with biotin-conjugated goat anti-mouse IgG (Invitrogen Corporation, CA, USA) for 40 min at room temperature. After washing in PBS, the sections were incubated with streptavidin-conjugated peroxidase (Invitrogen Corporation, CA, USA) 30 min at room temperature. After PBS washing, the sections were incubated with DAB (Invitrogen Corporation, CA, USA) followed by examination under the microscope.

### Senescence-associated β-galactosidase staining

Both the cells which were washed three times with PBS and cryostat sections (4 μm) which were mounted onto glass slides were fixed with 2% form-aldehyde and 0.2% glutaraldehyde for 5 min at room temperature and 10 min at 4°C. Next, the cells and cryostat sections were incubated for 14h at 37°C (without CO_2_) with freshly prepared senescence-associated β-galactosidase (SA-β-gal) staining solution (1 mg/ml X-gal, 40 mM citric acid/sodium phosphate [pH 6.0], 5 mM potassium ferrocyanide, 5 mM potassium ferricyanide, 150 mM NaCl, and 2 mM MgCl_2_). The cells were then washed with PBS, and 300–400 cells in six microscopic fields were counted to determine the percentage of SA-β-gal-stained positive cells; tissue sections were counterstained with eosin, 6 random fields per rat at a total magnification of 200 under a microscope.

### Immunofluorescence staining

Human PTCs were seeded into six-well plates with sterile coverslips at the bottom and according to different stimulation, they were cultured for 24h-72h. Then, cells were fixed in 4% paraformaldehyde for 5 min at room temperature and 10 min at 4°C, washed with PBS three times and permeabilized with 2% Triton X-100. After blocking with 5% bovine serum albumin (BSA) for 30 min, the cells were incubated with anti-E-Cadherin antibody (1:50 diluted with 5% BSA) and anti-α-SMA antibody (1:100 diluted with 5% BSA) at 4°C overnight. After washing with PBS three times, anti-E-Cadherin and anti-α-SMA antibodies were probed with fluorescein isothiocyanate-conjugated anti-mouse IgG (1:50 diluted with 5% BSA) and CY3-conjugated anti-rabbit IgG (1:200 diluted with 5% BSA) respectively for 1h in the dark. After washing with PBS three times, immunoblotted proteins were observed by fluorescence microscope.

### Renal histopathological studies

Kidney slices were embedded in paraffin after automated dehydration through a graded alcohol series, sections of 4μm thickness were prepared and stained with Masson’s trichrome staining. The sections were examined in a blind manner.

### Western blot analysis

Kidney tissues or cultured cells were lysed in radio immunoprecipitation assay buffer [50 mM Tris–Cl (pH 7.6), 150 mM NaCl, 1% NP-40, 0.1% sodium dodecyl sulfate (SDS), 0.5% deoxycholic acid, 1μg/ml leupeptin, aprotinin and antipain and 0.5 mM phenylmethylsulfonyl fluoride). Protein concentration was determined with the Pierce BCA assay kit (Lot#JK126465; Thermo Fisher Scientific, Rockford, IL, USA). Cells proteins in quantities of 50-80μg or tissues proteins in quantities of 120-200μg were separated by 8%–12% SDS-PAGE. Then transferred to an nitrocellulose membrane, subsequently probed with the following primary antibodies (diluted with CASEIN) at 4°C overnight: rabbit monoclonal anti-p21 at 1:500, anti-α-SMA at 1:500, anti-HIF-1α at 1:1000, anti-PPARα at 1:200, anti-P53 at 1:500; mouse monoclonal anti-E-cadherin at 1:1000, anti-vimentin at 1:2500, β-actin at 1:10000. After being washed with TBS containing 0.1% Tween 20 (TBST), Western blots were subsequently probed with horseradish peroxidase-conjugated anti-mouse, anti-rabbit IgG (Santa Cruz Biotechnology) at 1:2000. The bands were visualized by an enhanced chemiluminescence (ECL) system and recorded by an UVP Chemi Doc-It™ 600 Image system (Ultra-violet products Ltd, Cambridge, UK), and densitometry was performed using Quantity One software (Bio-Rad Laboratories, Hercules, CA, USA).

### EVs isolation

EVs were isolated from cell culture medium by differential centrifugation according to previous publications [6]. After centrifugated at 300×g, 1200×g, and 10,000×g for removing cells and other debris, the supernatant was centrifuged at 110,000×g for 2 hr (all steps were performed at 4°C). MVs were collected from the pellet and resuspended in PBS. The morphology of MVs was observed under transmission electron microscope (FEI TECNAI SPIRIT) [35]. Total RNA of MVs derived from cells was then extracted using TRIzol LS reagent (Invitrogen).

### Fluorescence labeling of EVs

HPTCs cells were labeled with Dil-C18 (Beyotime, Shanghai, China) for 1 hour and then washed five times with PBS in order to remove remaining Dil-C18. Harvesting EVs prepared as mentioned above, EVs were resuspended in DMEM/F12 medium and incubated with cultured recipient HPTCs cells. After incubation for 6h, cells were washed with PBS, fixed, and observed fluorescence microscope.

### Real time PCR for miRNAs expression

Total RNA from cells, EVs and tissues were extracted using TRIzol LS reagent according to the manufacturer’s instructions. Briefly, cDNA was synthesized, total RNA was reverse-transcribed to cDNA and the stem-loop reverse transcription primer were using AMV reverse transcriptase (Takara, Dalian, China). Quantitative PCR was performed with miR specific primers and miScript SYBR Green PCR Kit (Takara, Dalian, China) on 7500 Fast Real-Time PCR System (Bio-Rad). U6 snRNA was used as an internal control of cells or tissues. The ratio of miRNAs was calculated by using the equation 2^-ΔCT^. While, there was no template controls for EVs, so we calculate the absolute expression levels of target miRNAs, the CT values were calculated by referring to the standard curve which a series of synthetic miRNA oligonucleotides at known concentrations reversed transcribed and amplified to generate. All reactions were run in triplicate.

### Transfection of siRNA or miRNA

HPTCs cells (10×10^5^) were seeded in six-well plates and cultured for 18h to 40–50% confluence. 50 nmol/L Small interference RNA (siRNA)-HIF-1α (Santa Cruz, CA, USA) was mixed with 16μL transfection reagent (INTERFERin®) and diluted with 200 μL transfection buffer (OPTi-MEM). While for miRNA Transfection, 50 nmol/L miR-21 mimic or 100 nmol/L antisense miR-21 inhibitor or 50 nmol/L control miRNA (GeneCopoeia, Guangdong, China) was mixed with 8μL transfection reagent (jetPRIME™) and diluted with 200μL transfection buffer (jetPRIME™ buffer). After incubation for 15 min at room temperature, the mixture was added to the 2 mL DMEM/F12 medium, transfection medium replaced by new DMEM/F12 medium 24h after transfection and the cells were cultured for an additional 24 or 48h.

### miR-21 in fluorescent in situ hybridization (FISH)

miR-21LNA probes were purchased from Exiqon. Rats renal biopsies were fixed in prehybridization solution for 2 hours at 50°C, covered with plastic cover slip, and placed in a moist chamber. Then miR-21LNA probes and control probes were applied respectively. Hybridize overnight (18 hours) at 50°C with probes at the concentration of 25nM (1μL 2.5μM DIG labeled probe to 100μL hybridization solution), cover with plastic coverslip and place in a moist chamber. After wash, mouse anti-DIG (1:100), HRP-conjugated goat anti-mouse IgG (1:500), Cy5-tyramide working solution, and mounting medium with DAPI were used successively to visualize the positive hybridization signals. After overnight solidification, slides are ready for observation with fluorescent microscopy.

### Statistical analysis

Results are expressed as the means ± SD. Analyses were performed with SPSS 17.0 (SPSS Inc, Chicago, IL). The data which meet a normal distribution were analyzed with ANOVA, while those that did not fit the normal distribution were analyzed with a nonpara-metric Mann–Whitney test. P<0.05 was considered significant.

## Supplementary Material

Supplementary Figures
