# Supplementary material for: Caloric restriction alleviates aging-related fibrosis of kidney through downregulation of miR-21 in extracellular vesicles
Source: Aging (Albany NY). 2020 Aug 27;12(18):18052–72. doi: 10.18632/aging.103591 (PMC7585074; doi:10.18632/aging.103591)
Supplement: Supplementary Figures [file aging-12-103591-s001..pdf]

SUPPLEMENTARY FIGURES

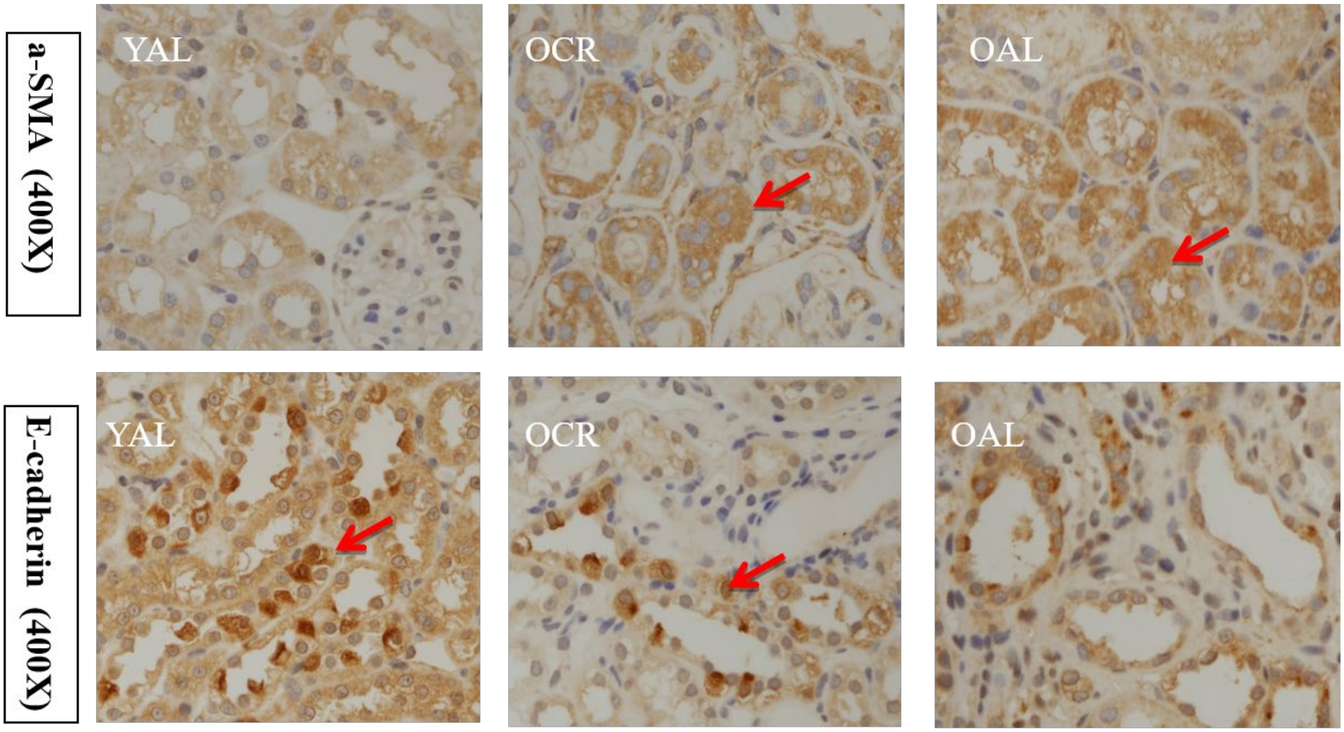

Supplementary Figure 1. Detection of E-cadherin, α-SMA level by immunohistochemistry staining in three groups.

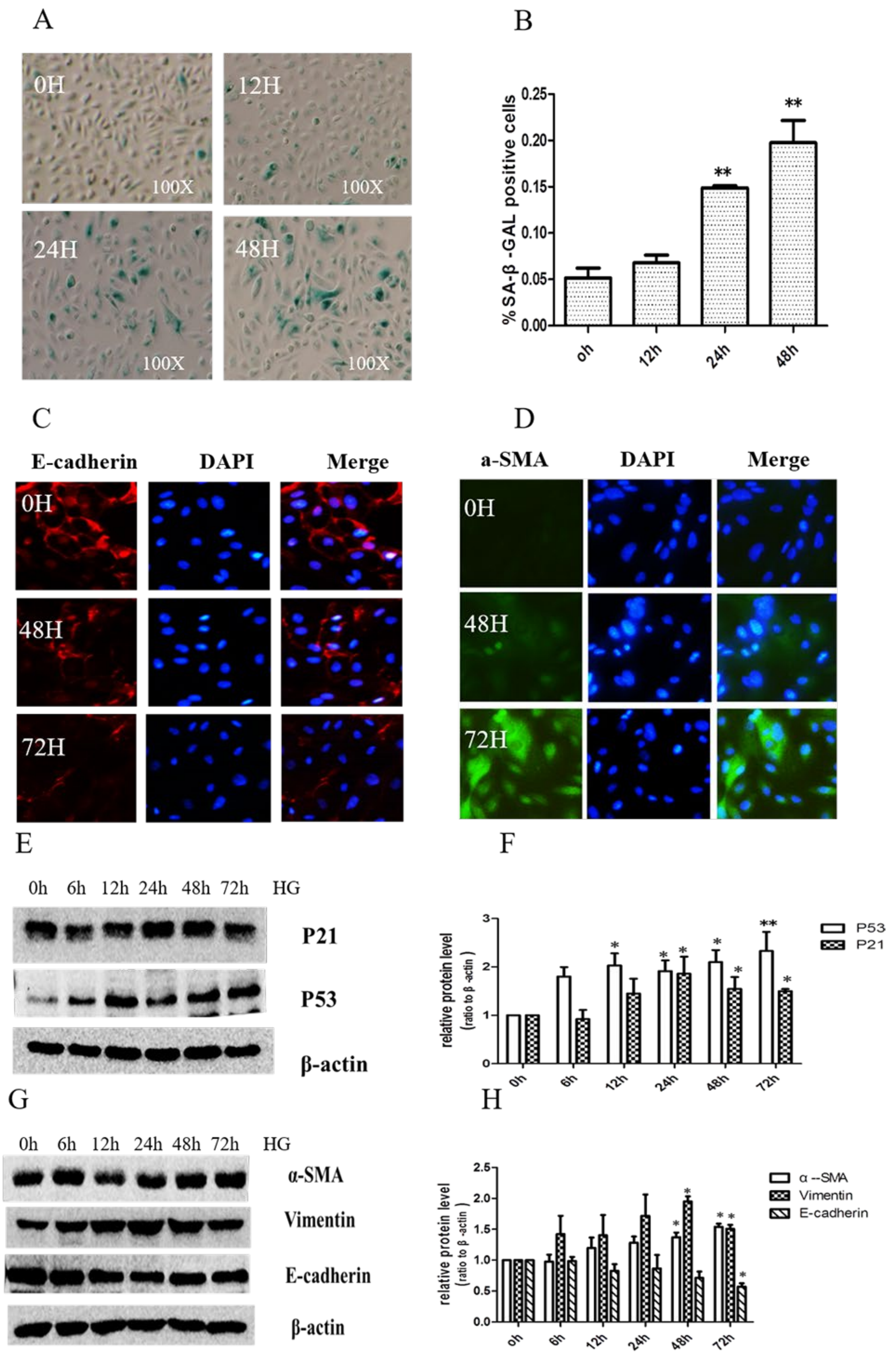
A
0H
12H
24H
48H
100X
B
% SA-β -GAL positive cells
0.25
0.20
0.15
0.10
0.05
0.00
**
0h
12h
24h
48h
C
E-cadherin
DAPI
Merge
0H
48H
72H
D
a-SMA
DAPI
Merge
0H
48H
72H
E
0h 6h 12h 24h 48h 72h HG
P21
P53
β-actin
F
relative protein level (ratio to β -actin)
P53
P21
G
0h 6h 12h 24h 48h 72h HG
α-SMA
Vimentin
E-cadherin
β-actin
H
relative protein level (ratio to β -actin)
α --SMA
Vimentin
E-cadherin

Supplementary Figure 2. The expression of E-cadherin, α-SMA, and vimentin expression. HPTCs senescence induced by high glucose. (**A**) Blue precipitation in cytoplasm was observed in HPTCs (100 magnification). (**B**) Quantitative analysis of SA- β-gal staining positive area from 6 random fields. ** $p < 0.01$. (**C**, **D**) Cells of each group were immunostained with anti-α-SMA and anti- E-cadherin at 0h, 48h, 72h after high glucose treatment (magnification, × 200). (**E**, **G**) Expression of the markers of senescence and EMT in HPTCs cultured in high glucose at 0h, 6h, 12h, 24h, 48h, 72h were quantified by Western blot (n=4). (**F**, **H**) Quantitative analysis of band density for the markers. The protein expression data are presented as mean±SD. * $p < 0.05$; ** $p < 0.01$.

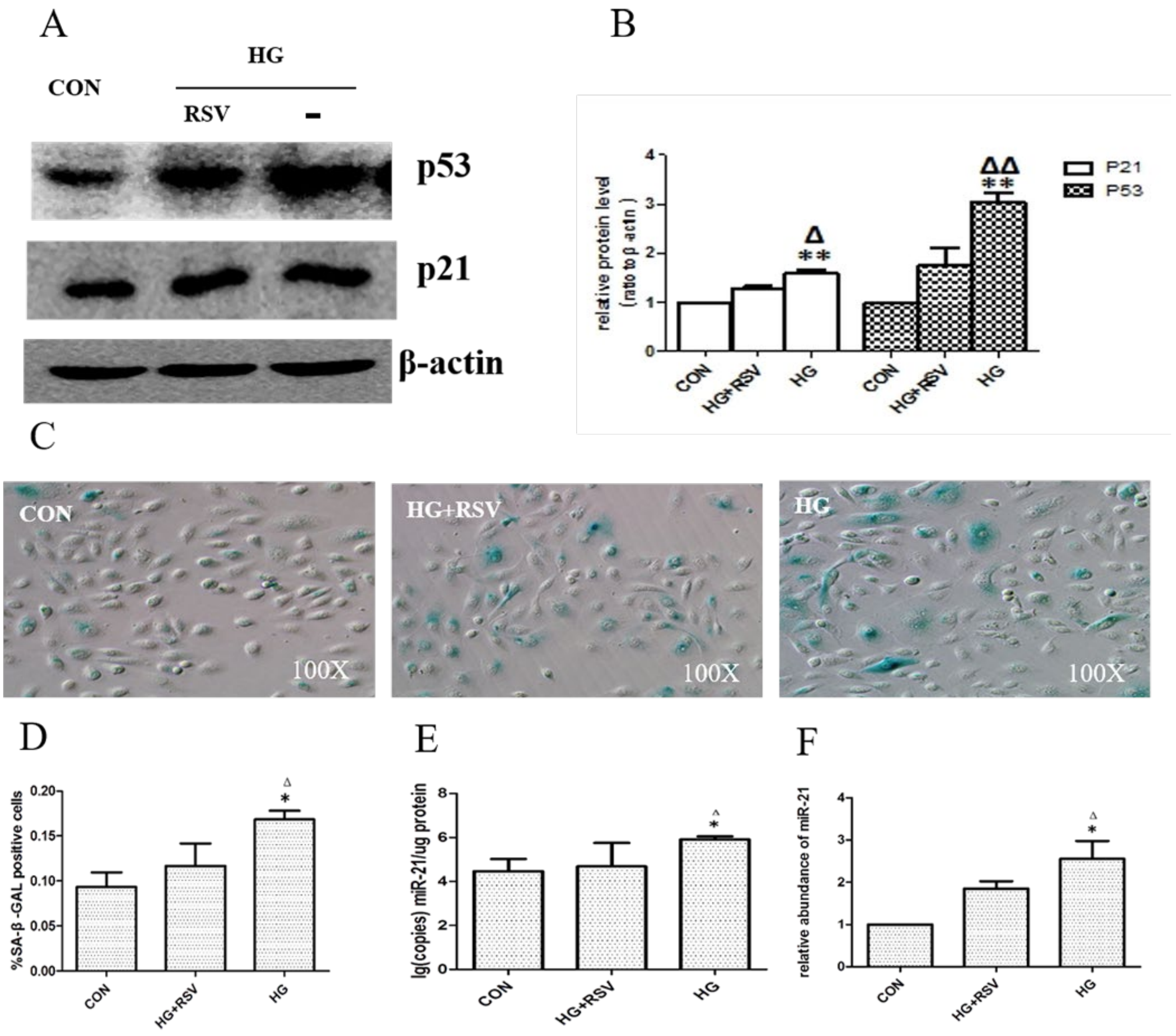

Supplementary Figure 3. The effects of high glucose and resveratrol on senescence phenotype and miR-21 expression. (**A**) The expressions of p21 and p53 protein were detected by Western blot analysis. (**B**) The ratios of p21 and p53 bands were analyzed. (**C**) SA-β-gal staining in HPTCs cultured by CON, HG, HG+RSV. Blue precipitation in cytoplasm was observed in the senescent cells (100 magnification). (**D**) Quantitative analysis of SA- β-gal staining positive area from 6 random fields. (**E**) Expression levels of EVs miR-21 in the three groups detected by qRT-PCR. (**F**) Expression levels of miR-21 in the three groups detected by qRT-PCR. * $p < 0.05$ (HG vs CON); Δ$p < 0.05$ (HG vs HG+RSV).
